# Costs and cost-effectiveness of three point-of-use water treatment technologies added to community-based treatment of severe acute malnutrition in Sindh Province, Pakistan

**DOI:** 10.1080/16549716.2019.1568827

**Published:** 2019-03-19

**Authors:** Eleanor Rogers, Hannah Tappis, Shannon Doocy, Karen Martínez, Nicolas Villeminot, Ann Suk, Deepak Kumar, Silke Pietzsch, Chloe Puett

**Affiliations:** a Independent Consultant, UK; b Department of International Health, Johns Hopkins Bloomberg School of Public Health, Department of International Health, Baltimore, MD, USA; c Operations department, Action Against Hunger USA, New York, NY, USA; d Programme department, Action Against Hunger Pakistan, Islamabad, Pakistan

**Keywords:** Therapeutic feeding programmes, community-based management of acute malnutrition; severe acute malnutrition, point-of-use water treatment, cost-effectiveness

## Abstract

**Background**: Severe acute malnutrition (SAM) is a major global public health concern. Despite the cost-effectiveness of treatment, ministries of health are often unable to commit the required funds which limits service coverage.

**Objective**: A randomised controlled trial was conducted in Sindh Province, Pakistan, to assess whether adding a point of use water treatment to the treatment of SAM without complications improved its cost-effectiveness. Three treatment strategies – chlorine disinfection (Aquatabs); flocculent disinfection (Procter and Gamble Purifier of Water [P&G PoW]) and Ceramic Filters – were compared to a standard SAM treatment protocol.

**Methods**: An institutional perspective was adopted for costing, considering the direct and indirect costs incurred by the provider. Combining the cost of SAM treatment and water treatment, an average cost per child was calculated for the combined interventions for each arm. The costs of water treatment alone and the incremental cost-effectiveness of each water treatment intervention were also assessed.

**Results**: The incremental cost-effectiveness ratio for Aquatabs was 24 US dollars (USD), making it the most cost-effective strategy. The P&G PoW arm was the next least expensive strategy, costing an additional 149 USD per additional child recovered, though it was also the least effective of the three intervention strategies. The Ceramic Filters intervention was the most costly strategy and achieved a recovery rate lower than the Aquatabs arm and marginally higher than the P&G PoW arm.

**Conclusions**: This study found that the addition of a chlorine or flocculent disinfection point-of-use drinking water treatment intervention to the treatment of SAM without complications reduced the cost per child recovered compared to standard SAM treatment. To inform the feasibility of future implementation, further research is required to understand the costs of government implementation and the associated costs to the community and beneficiary household of receiving such an intervention in comparison with the existing SAM treatment protocol.

## Background

Severe acute malnutrition (SAM) is a major public health concern in low- and middle-income countries. Over 16 million children suffer from the condition globally and it accounts for approximately 400,000 child deaths every year [1,2]. Treatment is provided through the Community-based Management of Acute Malnutrition (CMAM) model, of which one component is an outpatient service to treat cases without medical complications (cases presenting with complications receive inpatient care), made possible with the development of ready-to-use therapeutic food (RUTF), allowing the majority of treatment to be provided in the home [3].

This approach has been found to achieve comparable effectiveness and superior cost-effectiveness to the previously used inpatient model for the treatment of cases of SAM without complications. Rates of recovery from SAM, the primary measure of effectiveness, match those of inpatient care [3]. The cost per case recovered provides valuable information on the cost-effectiveness of SAM treatment; most of the existing evidence comes from soon after the development of the model, and has ranged from 145 US dollars (USD) in Ethiopia to 185 USD in Malawi [4,5]. Cost analyses that take a societal perspective, considering all costs for both the provider and the beneficiary household, estimated the outpatient model to also place less burden on beneficiaries, with treatment costing 6 USD (rounded) per child compared to 21 USD (rounded) for inpatient care in Ethiopia [4]. The majority of the evidence on cost-effectiveness of SAM treatment to date is from the African context although a study in Bangladesh, which employed a modified treatment strategy with community health workers (CHWs) delivering treatment to their own community, achieved a recovery rate of 91.9% and a cost per case recovered of 180 USD, similar to that of outpatient treatment in Malawi [6].

The cost per case treated has demonstrated a wider range, from 84 USD in Niger (2015) to 805 USD in Ghana (2012) [4–9]. The lower cost in Niger suggests that the cost-effectiveness of treatment may have improved in some contexts since its inception, for well-established programmes with a high caseload [8]. However the higher cost from Ghana reflects a setting with a high geographical coverage but low caseload (n = 40) resulting in a cost per child treated nearly 10 times higher than the former [9]. Global implementation contexts can vary considerably based on factors such as type of implementing agency, geographical coverage, caseload, treatment effectiveness, staff training requirements and RUTF costs.

Despite the cost-effectiveness of community-based SAM treatment, ministries of health (MoH) are often unable to commit the funds for the relatively high cost of treatment which continues to limit service coverage globally. One of the most costly inputs is RUTF, ranging from 13–44% of total costs, with the lowest figure from the Ghanaian study which treated only 40 cases. Local RUTF production and the development of less costly formulas are among strategies being explored to reduce costs [10–12]. Equally there are attempts to improve treatment effectiveness, such as a reduced dosage protocol [13]. Questions have also been raised about the effectiveness of treatment if children continue to use unclean drinking water [14]. An estimated 1.8 billion people worldwide use a faecally contaminated drinking water source, which can introduce pathogens leading to diarrhoea [15], which likely slows recovery from SAM.

Household-based point-of-use (PoU) water treatment interventions are an effective and cost-effective intervention to avert diarrhoea [16,17], even in the absence of improved water supply and sanitation [18]. Three key treatment methods are flocculent disinfectant, chlorine disinfectant and filtration. Flocculent-disinfectant water treatment has been found to be effective, reducing diarrhoea incidence by 90% in Liberia [19], and diarrhoea prevalence by 64% in Pakistan [20]. This treatment is effective even with turbid (cloudy) water [21]. Its performance, however, has varied regionally, with evidence indicating cost-effectiveness in East Africa but not in South East Asia [22]. Moreover it was the least cost-effective PoU method compared to chlorine disinfection, filtration and solar disinfection in a global study assessing data from 28 countries [22]. Chlorine disinfection has also demonstrated effectiveness in reducing the risk of diarrhoea [23], except in areas with high levels of turbidity [24,25]. An assessment of multiple water quality interventions found chlorine disinfection also to be the most cost-effective strategy over a range of settings [26,27]. Ceramic Filters are effective over long time periods [28,29] and while they are able to reduce faecal bacteria and protozoa, due to high implementation costs often they are found to be less cost-effective [26].

Despite potential for improvements in effectiveness and cost-effectiveness of SAM treatment, there is no evidence yet on the impact of the addition of a PoU water treatment. If this addition improved effectiveness and cost-effectiveness of SAM treatment, it would reduce the cost per case recovered, which, alongside other modifications to the treatment model, may make treatment more accessible for some ministries of health. To overcome this evidence gap, Action Against Hunger and Johns Hopkins University conducted a study in Sindh Province, Pakistan, to test whether the addition of PoU water treatment improved cost-effectiveness of the treatment of uncomplicated SAM for children aged 6–59 months. The objective of this analysis was to compare the cost-effectiveness of adding three different PoU household water treatments to a standard SAM treatment protocol, in terms of the incremental, or additional, cost to recover an additional child from SAM with each treatment compared to standard SAM treatment.

## Methods

### Description of the intervention

Between February and October 2016, in the predominantly rural Dadu district in Sindh Province, Pakistan, a four-group site randomised controlled trial (RCT) was conducted to test whether the provision of PoU water treatment and education on its use influenced the effectiveness of community-based outpatient treatment for cases of SAM without complications [30]. In 2014 SAM prevalence in Dadu was estimated to be 3.8% (95% CI 2.3%-6.4%), and although drinking water sources have improved considerably over the last 10 years, in Sindh 87.2% of the population living without an improved water source do not use an appropriate treatment method [31]. Action Against Hunger has delivered CMAM services in Dadu district since 2012 through health facilities under the responsibility of the Ministry of Health; the programme staffing structure is available in supplementary file 1.

Twenty eligible outpatient therapeutic programme (OTP) sites were randomly assigned to one of four groups based on the following criteria: (1) projected caseload, such that reasonably similar numbers of SAM cases would present to facilities in each group during the study period; and (2) urban/rural/remote OTP site location, such that proportions of children in the caseload coming from each of these environments would be as similar as possible across comparison groups. Locations of each group of facilities were subsequently mapped to ensure balanced spatial coverage and were reviewed by field staff to ensure similarity and logistical feasibility. After agreement on the assignment of OTP sites to four comparison groups, each group was randomly assigned to an intervention. All four study groups received SAM treatment which included health screening, regular growth monitoring and provision of RUTF, as well as the addition of hygiene education and water storage containers for household use (hereafter referred to as ‘standard SAM treatment’).

Each group also received one of the following water treatment interventions, chosen because they are products commonly recommended in household water treatment interventions, are locally or regionally available, in particular in Pakistan’s economic capital Karachi (also in Sindh, 300 km from Dadu District), and are relatively easy to use by households and to monitor by researchers: (1) chlorine disinfectant (referred to as ‘Aquatabs’ hereafter, a locally available mass market product); (2) flocculent-disinfectant water treatment (P&G Purifier of Water, referred to as ‘P&G PoW’ hereafter); and (3) ceramic water filters (locally available mass market product, referred to as ‘Ceramic Filters’ hereafter). A fourth ‘no intervention’ group acted as a control, and households continued with their routine approach to water treatment, if employed, with the addition of hygiene education and water storage containers to represent the conventional water source used by beneficiaries receiving SAM treatment and to enable comparison with the other study arms. Blinding was not possible due to the obviously different nature of the PoU interventions.

As outlined in a paper describing the RCT, the minimum (n = 200) and maximum (n = 300) sample size per arm was calculated based on the primary study outcome, length of stay [30]. A total of 901 children were enrolled in the study (219 at control sites, 231 at Aquatabs sites, 220 at Ceramic Filter sites and 231 at P&G PoW sites). Eligible children were aged between 6 and 59 months, diagnosed as an uncomplicated SAM case, enrolled in the SAM treatment programme at a participating site and had caretaker’s consent to participate. Baseline characteristics are available in the effectiveness paper [30]. Recovery rates, the secondary study outcome, were calculated based on participant status upon exit or after 120 days of enrolment, whichever came first, with participants classified as recovered when their mid-upper arm circumference (MUAC) was >12.5 cm for two consecutive weeks.

### Analytical strategy

The analysis used standard methods for cost-effectiveness analysis within Action Against Hunger (Puett C. (2019) Assessing cost-effectiveness of interventions within a humanitarian organization. *Disasters*, 43(3): In press.), with the exception of adopting a provider perspective, rather than including costs to beneficiary households and the wider community, due to cost data being collected remotely. The cost analysis therefore includes the direct and indirect costs incurred by the implementer, the non-governmental agency Action Against Hunger, and select costs from government sites for rent and utilities. This analysis includes costs incurred for the nine-month implementation period from February to October 2016, as well as those incurred in January during preparation, including staff time and associated office costs.

Costs were grouped according to the two programme components: (1) community-based standard SAM treatment underlying all four arms of the trial, and (2) the specific costs for PoU water treatment incurred in each arm. These two groups of costs were analysed as follows. First, costs for SAM treatment and water treatment were summed to estimate an average cost per child treated and per child recovered for the combined interventions (i.e. SAM treatment + water treatment) for each arm. Then the water treatment costs alone were analysed to find the average cost per child of the additional water treatment intervention in each arm. Last, the incremental cost-effectiveness of each intervention was assessed, defined by the additional cost per additional child recovered by the three PoU water treatments relative to the control arm, represented by standard SAM treatment with the inclusion of water storage containers and hygiene education, and related monitoring and logistics.

### Data collection

Costs for the household water treatment interventions were collected between May and August 2016. Thirty-four interviews with field, management, technical and support staff were conducted to discuss staff roles and activities in the project and to identify the proportion of time each staff member spent on implementation in the various water treatment interventions. Data collection was conducted remotely via teleconference.

### Cost analysis

Costs of SAM treatment were adapted from a recent cost analysis of the same program in the study area [32]. These costs were scaled for number of OTP sites (n = 5 per arm) and proportion of beneficiaries at each site who were included in the present study; cost of RUTF was calculated based on the number of children treated in each arm. All costs per arm for SAM treatment, except for the cost of RUTF, were divided by the proportion of OTP exits that participated in the RCT in each arm; this varied per arm from 35% to 47%. The cost of RUTF supply, storage and transport was then added.

The cost of implementing the water treatment interventions was determined through an assessment of accounting data provided by Action Against Hunger. Implementation costs were allocated to study arms based on estimates of time allocation from implementing staff. Costs related to implementation of programme activities were included. Research-related costs for the RCT were excluded. Start-up costs for the water treatment intervention are considered, but not for standard SAM treatment as it was an established programme at the time of the RCT. Total costs for the implementation of SAM treatment and for the implementation of each PoU water treatment, including related management and logistics, were calculated separately and summed to estimate the total cost of each arm.

Costs were regularly converted from Pakistani Rupees into USD in the provider accounting system and are presented in 2016 USD. Costs were not adjusted for inflation, as the intervention took place within a one-year period. Costs of the stabilisation centre were included in this analysis despite no inpatient outcomes, based on the assumption that SAM treatment for uncomplicated SAM cannot be delivered without making inpatient treatment available.

### Effectiveness data

Outcome data on recovery rates were taken from the RCT in Dadu District, defined as the proportion of children exiting the programme cured within 120 days of enrolment. Outcome data are presented in Table 3.

**Table 1.** Costs of SAM treatment for each study arm.

	**Control**		**Aquatabs**		**P&G PoW**		**Ceramic Filters**	
**Personnel**	**USD**	%	**USD**	%	**USD**	%	**USD**	%
CHWs *(volunteer incentives)*	709	2.0	582	1.8	792	2.1	671	2.0
Field staff *(community mobilisation, nutrition supervisors and OTP nurses)*	7,745	21.7	6,359	19.6	8,652	22.4	7,323	21.5
Management and technical staff *(coordinator, prog. manager & deputy)*	1,192	3.3	979	3.0	1,332	3.4	1,127	3.3
Stabilisation centre *(doctor, nurse and support)*	1,443	4.0	1,185	3.7	1,612	4.2	1,364	4.0
Support staff *(logistics, finance, administrative, guards)*	1,670	4.7	1,371	4.2	1,865	4.8	1,579	4.6
**Programme costs**								
Office materials	1,241	3.5	1,019	3.1	1,386	3.6	1,173	3.5
SAM treatment IEC materials	429	1.2	352	1.1	479	1.2	405	1.2
RUTF supply	13,749	38.5	14,330	44.3	14,072	36.4	13,233	38.9
**Logistics**								
Office *(rent and utilities)*	1,050	2.9	862	2.7	1,172	3.0	992	2.9
Warehouse *(rent and utilities)*	76	0.2	63	0.2	85	0.2	72	0.2
Health centres *(rent and utilities)*	1,815	5.1	1,491	4.6	2,028	5.3	1,716	5.0
Stabilisation centre *(rent and utilities)*	1,341	3.8	1,101	3.4	1,498	3.9	1,268	3.7
Transport *(car rental, maintenance and fuel)*	3,254	9.1	2,671	8.3	3,635	9.4	3,076	9.0
Total (USD)	35,714	100.0	32,364	100.0	38,609	100.0	33,999	100.0

Note: These costs are prorated for the proportion of children in the RCT in each study arm and do not reflect the total cost incurred for each input in the entire overarching SAM treatment programme.

**Table 2.** Cost of implementing the PoU water treatment interventions alone and combined costs of water treatment and SAM treatment.

	**Control**		**Aquatabs**		**P&G PoW**		**Ceramic Filters**	
	**USD**	%	**USD**	%	**USD**	%	**USD**	%
**Personnel**								
WASH field staff *(hygiene promotors, nutrition and hygiene supervisors)*	10,188	54.3	10,354	50.0	10,551	42.9	17,397	41.9
Programme management staff *(study manager and deputy)*	1,076	5.7	1,076	5.2	1,916	7.8	2,757	6.6
Technical advisors *(WASH engineer and general)*	735	3.9	831	4.0	1,244	5.1	1,021	2.5
Support staff *(logistics, finance, administrative)*	1,736	9.3	2,373	11.5	2,373	9.7	3,010	7.2
**Programme costs**								
Water storage containers	1,108	5.9	1,108	5.4	1,108	4.5	1,108	2.7
Water treatment materials	0	0.0	507	2.5	2,831	11.5	9,294	22.3
Water quality testing *(materials and cost of time required)*	0	0.0	459	2.2	459	1.9	396	1.0
IEC materials *(hygiene promotion and treatment guidance)*	267	1.4	315	1.5	315	1.3	315	0.8
**Logistics**								
Warehouse *(rent and utilities)*	910	4.9	910	4.4	910	3.7	910	2.2
Transport *(car rental, maintenance and fuel)*	2,730	14.6	2,792	13.5	2,866	11.7	5,428	13.0
**Total cost of water treatment**	18,750	100.0	20,725	100.0	24,573	100.0	41,637	100.0
**Additional cost compared to the control**	-	-	1,975	-	5,823	-	22,887	-
**SAM treatment + PoU water treatment**	54,464	-	53,088	-	63,182	-	75,636	-

**Table 3.** Effectiveness and cost outcomes for four study arms.

	Control	Aquatabs	P&G PoW	Ceramic Filter
Recovery rate	53.1%	75.2%^a^	69.7%^a^	70.7%^a^
Defaulted	12.7%	5.0%	7.3%	7.8%
Died	0.5%	0.5%	0.9%	2.0%
Non-recovered	33.0%	19.4%	22.0%	19.5%
# children treated	213	222	218	205
# children recovered	113	167	152	145
Cost of the combined interventions: SAM treatment + PoU water treatment	54,464	53,088	63,182	75,636
Cost per child treated (USD)	256	239	290	369
Cost per child recovered (USD)	482	318	416	522
Cost of the PoU water treatment component alone (USD)	18,750	20,725	24,573	41,637
Cost of the PoU water treatment per child treated (USD)	88	93	113	203
Cost of the PoU water treatment per child recovered (USD)	166	124	162	287

Note: ^a^All three intervention arms had significantly higher recovery rates compared to the control arm [30].

### Cost-effectiveness analysis

Costs and outcomes for all arms were modelled in TreeAge Pro 2016 software. Sensitivity analyses were conducted to determine the extent to which the results and conclusions of the analysis might change given plausible variation in study parameters related to costs and recovery rates. Univariate sensitivity analyses were conducted by deterministically varying parameters individually by ± 25% of the base case values, consistent with approaches in other settings [5,7].

Multivariate probabilistic sensitivity analyses assessed variation in all parameters simultaneously, using 1,000,000 replicates. Such analyses are based on assumptions about the distributions of costs and effects. Cost data often have skewed distributions; a gamma distribution was used to characterize the cost parameter, as it allows for high outliers associated with costs and is constrained to positive values. Beta distributions were chosen for recovery rates, as they are appropriate for modelling proportions.

## Results

### Costs: base case

Significant costs of the intervention were field staff (~20%), transport (~10%), and RUTF costs at 40%, 10% of which were due to import fees. Transport costs were high, as vehicles were predominantly used by field staff for visiting OTPs, communities and households as required for follow up. A full breakdown of costs for SAM treatment in each study arm is presented in Table 1.

A breakdown of the costs of the water treatment components in each arm are presented in Table 2.

Field staff was the largest cost across all arms. This cost is comprised of staff time allocation from hygiene promotors, hygiene promotor supervisors and nutrition supervisors, the last of which played a larger role in implementation of the delivery of the household water treatment compared to SAM treatment. Despite there being no water treatment intervention implemented for households in the control arm, there were large staff costs incurred in the control arm. This represents a close level of household monitoring in this arm of the use of the water storage containers as part of the standard SAM treatment package.

In all four arms, each household received one staff visit for household monitoring to ensure the correct use of water storage containers and/or treatment materials. Each household in the Ceramic Filters arm was visited an additional time for the delivery and set up of the filter itself, thus doubling the field staff and associated transport costs. The beneficiaries in the remaining three arms were able to carry their treatment materials and/or storage containers themselves, so these items were distributed at the OTP sites. Therefore, field staff costs in the control, Aquatabs and P&G PoW arms were similar but were much higher in the Ceramic Filters arm. More management and technical staff time was required in the three intervention arms, compared to the control, due to the support required for treatment product usage. Ceramic Filters required the most guidance in the beneficiary home, followed by the P&G PoW tablets, leading to higher management costs. Aquatabs required minimal additional guidance, with management costs comparable to the control group. The additional cost of management and technical staff between the control and the Aquatabs arm was minimal, at 95 USD.

Storage containers and IEC materials alone cost 1375 USD per arm, with additional materials costs incurred for water treatment in the three intervention arms. The most expensive treatment materials were the Ceramic Filters, with the P&G PoW materials a third of that amount and Aquatabs considerably less. For each of the three intervention arms the cost of one water quality test was included for each household, which cost less in the Ceramic Filters arm due to the type of test used. This cost was included in the analysis, as it is often a donor requirement for NGO-delivered programmes.

### Cost-effectiveness

#### Base case analysis

Table 3 presents the base case cost outcomes and average cost-effectiveness ratios for all study arms for the combined SAM treatment and water treatment interventions and for the water treatment interventions alone.

The cost of water treatment alone represents the cost of implementing water treatment and water storage interventions, and related management and logistics across all study arms. The costs of implementing SAM treatment are not included in these estimates; therefore, when used in the cost-effectiveness analysis they represent the cost of the water treatment intervention components alone as implemented in each arm.

Table 4 presents incremental cost-effectiveness ratios (ICER) per study arm, defined as the additional cost incurred per additional child recovered by the three PoU water treatments compared to the control arm. The analysis in column F reflects the comparison of costs and effectiveness of each study arm with the control group only, since differences in recovery rates between study arms were not statistically significant.

**Table 4.** Incremental cost-effectiveness of all strategies relative to control arm.

A. Strategy	B. Additional cost per child of water treatment	C. Incremental cost	D. Effectiveness (recovery rate)	E. Incremental Effectiveness relative to control	F. ICER relative to control arm
Control	88	-	0.53	-	-
Aquatabs	93	5.33	0.75	22.1%	24
P&G PoW	113	24.69	0.70	16.6%	149
Ceramic Filters	203	115.08	0.71	17.6%	654

Notes: ICER = incremental cost-effectiveness ratio reflecting additional cost per additional child recovered in each intervention relative to the control group; Calculated as (cost arm 1 – cost arm 2)/(effectiveness arm 1 – effectiveness arm 2) or (incremental cost/incremental effectiveness).

In comparison with the control arm, the next least expensive option was Aquatabs, which was also the most effective strategy. The ICER for Aquatabs was 24 USD, signifying the additional cost incurred for each additional child recovered who received the Aquatabs intervention compared to the control arm. The P&G PoW arm was the next least expensive strategy, though it was also the least effective of the three intervention strategies. Finally, the Ceramic Filters arm was the most costly strategy, achieving a recovery rate lower than the Aquatabs arm and marginally higher than the P&G PoW arm.

In this base case analysis, the clear dominant strategy was the Aquatabs intervention, being cheaper and more effective than the other household water treatments. P&G PoW and Ceramic Filters were absolutely dominated, being both more costly and less effective than Aquatabs.

All three intervention arms achieved similar recovery rates, within five percentage points of one another and with no significant differences between arms [30]. Further, the costs per child treated in each arm as shown in Table 3 were similar, particularly for the Aquatabs and P&G PoW arms. This similarity suggests that any potential variation in the costs and outcomes from the base case presented here may change this study’s conclusion as to which was the most cost-effective intervention. This question was explored further in sensitivity analyses.

### Sensitivity analysis

Table 5 presents parameter values and ranges used in the sensitivity analyses.

**Table 5.** Model parameter values and ranges.

Parameter	Base case	Worst case	Best case	Distribution	Source
Recovery rate, control	53.1%	39.8%	66.3%	Beta	Base: RCT results Worst: −25% of base case Best: +25% of base case
Recovery rate, Aquatabs	75.2%	56.3%	93.9%		
Recovery rate, P&G PoW	69.7%	52.1%	86.9%		
Recovery rate, Ceramic Filters	70.7%	53.2%	88.6%		
Cost per child, control	88	110	66	Gamma	Base: average cost per child of the addition of water treatment Worst: +25% of base case Best: −25% of base case
Cost per child, Aquatabs	93	117	70		
Cost per child, P&G PoW	113	141	85		
Cost per child, Ceramic Filters	203	254	152		

### Univariate sensitivity analysis

Based on results from the base case analysis, several variables were explored in more depth in univariate sensitivity analysis. This analysis focused on the costs and effects of the Aquatabs and P&G PoW arms to determine to what extent variation over a plausible range of values (± 25% of the base case) for each variable influenced which intervention was more cost-effective.

In the analysis of the Aquatabs arm, varying the recovery rate over its plausible range resulted in a change in ICER from 13 to 167 USD. Aquatabs had a higher recovery rate than the control arm across all plausible values. Further, it dominated the P&G PoW and Ceramic Filters arms due to better effectiveness in over half of this plausible range of values, specifically when achieving recovery rates higher than P&G PoW and Ceramic Filters (70–71%). When varying the cost of Aquatabs, at the lower end of its plausible range it dominated all other arms due to being the cheapest and most effective strategy. At its highest plausible cost (117 USD), it no longer dominated P&G PoW due to being slightly more expensive while remaining more effective.

For the analysis of the P&G PoW arm, the recovery rate showed the most variation in the model; varying this over its plausible range resulted in a change in ICER relative to Aquatabs ranging from −84 to 166 USD. The negative outcomes from this analysis indicate that at the low end of its range of possible effectiveness the P&G PoW arm was dominated by both Aquatabs and the control arm, which had better effectiveness and lower costs. When varying recovery rates achieved by Aquatabs (75%), the P&G PoW arm was no longer dominated by the Aquatabs arm, though it remained more costly. When varying the cost of the P&G PoW arm, it remained dominated by Aquatabs except at the lowest end of its range, while continuing to be less effective.

In summary, when assessing relative cost-effectiveness of Aquatabs and P&G PoW by varying one parameter at a time, in most cases Aquatabs dominated P&G PoW, being both less costly and more effective; there was never an instance where P&G PoW dominated Aquatabs. This strong domination indicates that in most cases Aquatabs was the preferred strategy, being more cost-effective relative to P&G PoW.

### Probabilistic sensitivity analysis

**Figure 1.**
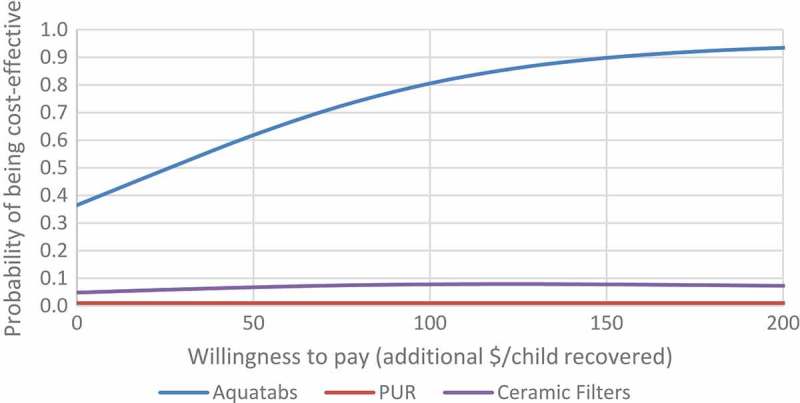
Cost-effectiveness acceptability curves for all PoU water treatments.

Figure 1 presents cost-effectiveness acceptability curves, representing the probability of each arm being considered cost-effective at different levels of willingness to pay to recover a child from SAM, up to a limit of 200 USD. The willingness to pay value presented here is arbitrary and was selected to show a wide range of probabilities for each arm to be considered cost-effective. The curves represent incremental costs and outcomes of each arm relative to the control; therefore, these results indicate the probability of each arm being more cost-effective than standard SAM treatment, as represented by the control arm, in this setting.

The figure shows that even at a willingness to pay of zero there is a 35% likelihood of Aquatabs being cost-effective compared to standard SAM treatment; this is due to the relatively low incremental cost per child recovered for Aquatabs (24 USD), representing the additional cost of this strategy when added to standard SAM treatment as represented in the control arm. The probability of Aquatabs being cost-effective was 75% at a willingness to pay of 86 USD per additional child recovered, and reached a 90% probability at a willingness to pay of 160 USD per additional child recovered. The 95% confidence interval for the incremental cost-effectiveness ratio of Aquatabs relative to the control arm was −126 to 184 USD.

As was found in the univariate sensitivity analysis, the probabilistic analysis found both P&G PoW and Ceramic Filters to be absolutely dominated by the Aquatabs strategy, as they were more costly and less effective. While the P&G PoW and Aquatabs strategies were similar in cost and recovery rate, the probabilistic analysis found that there was less than a 10% probability of P&G PoW being more cost-effective than Aquatabs. The 95% confidence interval for the ICER of P&G PoW relative to the control arm was −62 to 488 USD.

Findings for the Ceramic Filters were the same in the probabilistic analysis as in the univariate analysis, in that they were not likely to be cost-effective under any scenario, given their high costs and low effectiveness relative to the other intervention options. The 95% confidence interval for the ICER of Ceramic Filters relative to the control arm was 315 to 1482 USD.

## Discussion

This study assessed the costs and cost-effectiveness of SAM treatment with the addition of one of three point-of-use (PoU) household water treatments, compared to standard SAM treatment with provision of water storage containers and hygiene education in Dadu district, Pakistan. When the PoU household water treatment component was added to standard SAM treatment, effectiveness increased, and although it also increased implementation costs, the average cost per child recovered was lower for the Aquatabs and P&G PoW arms than for the control arm. In assessing the incremental cost-effectiveness of the three water treatments relative to standard SAM treatment, considering only the additional costs of water treatment and excluding costs related to SAM treatment in all arms, Aquatabs was the most cost-effective strategy at 24 USD per additional child recovered relative to standard SAM treatment. Care is required, however, in assuming replicability of these results with a focus on simple chlorine disinfection (represented by Aquatabs), as its effectiveness in treating turbid water or protozoa is limited; the choice of a treatment method needs to take into account the context and water characteristics [33]. There is no evidence from other settings for direct comparison of the additional cost of water treatments to recover a child from SAM as this is a new area of research.

The primary outcome of the effectiveness study was length of stay in SAM treatment, which assessed whether this would be reduced with the addition of clean drinking water, leading to less RUTF required per child treated. However, there was no difference in the average length of stay, either between the control and the three treatment groups or between the three treatment groups themselves [30], so the cost savings sought have not materialised. Data on the cost-effectiveness of other interventions to increase the effectiveness of SAM treatment have not yet been published, so no comparisons can be made with any alternative approaches aiming to reduce treatment cost to increase global coverage.

### Costs

As with all costing data, it is critical to consider the implementation context, and in this study there were a number of factors that may have driven up costs. First, the programme was delivered by an NGO and had a high level of management and technical support, as well as guidance to beneficiaries on product use, as it was part of an RCT. The inclusion of the control households in implementation staff visits meant that the control arm, in which households did not receive a water treatment intervention, still incurred significant costs for this programme component. Second, the two treatment components were delivered by separate field teams, and although conservative assumptions were made about subsuming overlapping costs, it is likely that a more efficient approach could be found in practice. Third, as staff and the beneficiary community became familiar with treatment materials there would be less time burden on staff, creating cost efficiencies. For these reasons, it is likely that these results are a conservative estimate for both SAM treatment and the water treatment components, and in practice these costs could be reduced. Further analysis in a more routine operational setting outside of a study context would be of value to inform future implementation.

The largest difference in the implementation cost of each PoU water treatment method was the cost of treatment materials and the associated staff time and resources required to support households to use the products as directed. Compared to all of the other options tested, Aquatabs cost the least, had minimal transport costs for the programme and required little guidance for use, so the cost of providing Aquatabs as an addition to the hygiene education and storage containers component measured in the control arm was minimal.

The cost of testing the water quality in a sample of households by the implementing agency was included in this analysis, considered good practice to ensure that the treatment provided is being used appropriately. This cost, however, which amounted to approximately 300 USD per arm, could be eliminated if such services were implemented by a national government with limited resources.

Cost-effectiveness is not the only consideration when choosing a PoU water treatment intervention [26]. The cost in time and money and any opportunity cost incurred by beneficiaries, along with user preference, influencing the sustainability of the approach, require consideration but were not taken into account in this study. Challenges around the sustainable use of PoU water quality interventions is well documented [34], and in other contexts the health benefits gained, such as the reduction of diarrhoea, from the use of effective water treatment and the cost to the household were not always determining factors for use [35,36]. Access to the product and its cost to the beneficiary have been recognised as contributing factors, but acceptability, in particular of taste of water, ease of use, and habits and beliefs are some of the barriers challenging adequate adherence to any water treatment. A better understanding of societal costs is therefore necessary as the costs to, and preferences of, end users could influence a decision as to the most appropriate intervention.

### Generalisability

Although this NGO-supported programme in Sindh province resulted in high costs, which may be too high to encourage replication, there is potential for the PoU water treatments to improve the recovery rate of standard SAM treatment. Evidence on implementation of the approach by the government only would be beneficial as it may result in cost reductions.

### Limitations

This analysis has two key limitations. First, this study did not adopt a societal perspective, thus limiting our understanding of the impact of the intervention on communities and therefore of the likelihood of long term sustainability outside of a research context. Second, the recovery criteria used at 12.5 cm and 120 days, in line with international standards but stricter than the Pakistan national protocol for treatment of SAM, may have contributed to higher non-response rates and lower recovery rates in this study compared to previous programme data from the same district and other contexts [30].

## Conclusion

This study found that the addition of a point-of-use drinking water treatment intervention to the treatment of uncomplicated SAM improved cost-effectiveness outcomes compared to the provision of standard SAM treatment, represented by SAM treatment, water storage containers and hygiene education, in this setting. Of the three water treatments tested, chlorine disinfection (Aquatabs) was the most cost-effective, with the highest effectiveness and lowest costs compared with flocculent disinfection (P&G PoW) and Ceramic Filters. To inform the feasibility of future implementation, however, further research is required to understand the costs of government implementation and the associated costs to the community and beneficiary household of receiving such an intervention in comparison with standard SAM treatment.
