# Supplementary material for: Costs and cost-effectiveness of three point-of-use water treatment technologies added to community-based treatment of severe acute malnutrition in Sindh Province, Pakistan
Source: Glob Health Action. 2019 Mar 19;12(1):1568827. doi: 10.1080/16549716.2019.1568827 (PMC6427553; doi:10.1080/16549716.2019.1568827)
Supplement: Supplemental Material [file ZGHA_A_1568827_SM6270.docx]

Supplementary file 1

Programme staffing structure, an outline of the staff positions that were involved with the implementation of the intervention in Dadu District, Pakistan.

Table 1: Programme staffing structure

|  | **SAM treatment (per arm)** | **PoU water treatments (shared across 4 arms)** |
| --- | --- | --- |
| Field | OTP:   - OTP nurses - Nutrition supervisors - Community Mobilisers - IYCF promotors - Capacity building officer   Stabilisation Centre:   - Doctor - Nurse | - Hygiene Promotion and Data Gatherers (HPDGs) - Nutrition Supervisors - Hygiene Promotion Supervisors |
| Management and technical | - Project coordinator - Nutrition Programme manager - Deputy Nutrition Programme Manager | - WASH Specialist - Technical Manager - Manager - Deputy Manager |
| Support | - Human Resources - Logistics - Finance - Stabilisation Centre support | - Human Resources - Logistics - Finance |
